# Supplementary material for: Simulated synapse loss induces depression-like behaviors in deep reinforcement learning
Source: Front Comput Neurosci. 2024 Nov 6;18:1466364. doi: 10.3389/fncom.2024.1466364 (PMC11576168; doi:10.3389/fncom.2024.1466364)
Supplement: Supplementary file 1 [file Data_Sheet_1.pdf]

Model parameters

Deep Q Learning parameters for “healthy” agent

Neural network architecture:	100 input neurons, 10 hidden neurons, 3 output neurons
Optimizer:	adam optimizer with mean-square-error loss function
Replay buffer size:	10,000 experiences
Network updates:	Every 5 steps, based on 1,500 experiences sampled from buffer
Learning rate:	0.01
Weight decay:	0
Discount factor (γ):	0.9
Exploration rate (ε):	0.05

Parameters for simulated spine loss

Increase weight decay to 3E-3
This value was chosen (somewhat arbitrarily) using the results in figure 7

Parameters for reduced RPE model

Reduce learning rate to 0.001 (equivalent to scaling RPE by 0.1)

Parameters for higher negative learning rate model

Multiply positive rewards by 0.5. Multiply negative rewards by 2.

Parameters for high discounting model

Decrease γ to 0.5

Parameters for high exploration model

Switch from ε-greedy action selection to softmax-based selection with τ=1

Observing responsivity of neurons through neuron bias

It is typical to apply weight decay to artificial neural networks that affects network weights and biases equally. Here, to investigate the effect of weight (only) decay on the network biases, we apply large weight decay of 3E-3 (as in our other simulated spine loss experiments) to only the weights of the “depressed” agents neural network. To the biases we apply a separate, more mild and more typical weight decay of 1E-5. This agent and a healthy agent were allowed to learn in the simulated environment as in the other experiments. After learning, the “depressed” agent’s neuron biases were all much higher than the “healthy” agent’s biases. The larger biases indicate more sensitive firing thresholds for the “depressed” neurons; likely an attempt to compensate for the reduction in input signal caused by simulated spine loss.

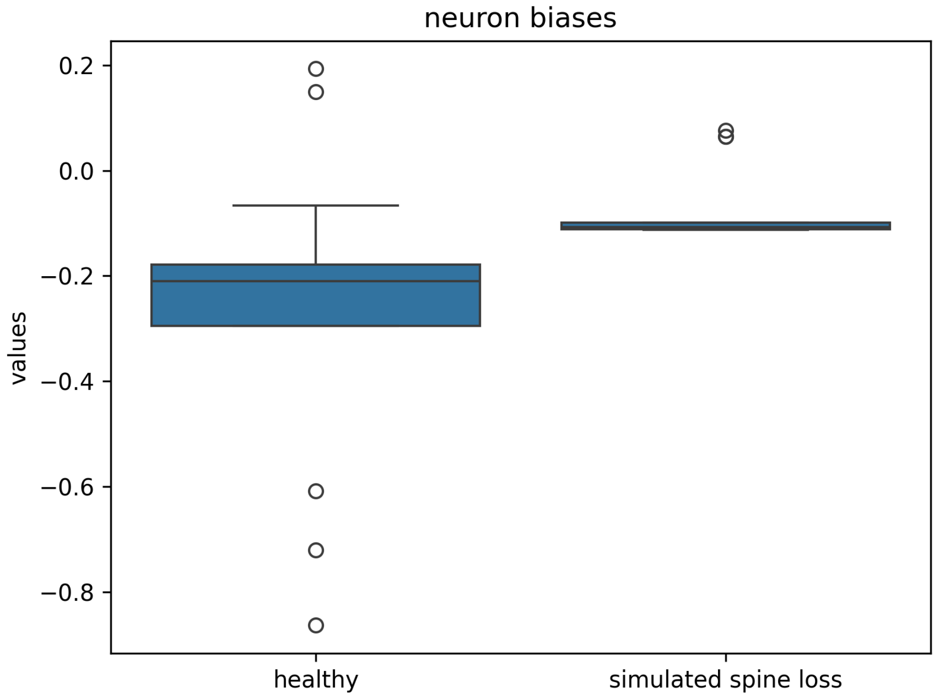

Figure S1, When weight decay is applied to connection weights between neurons, the network attempts to compensate by increasing neuron biases. This can be interpreted as an increased sensitivity to excitation.

Simulating spine loss by deleting neural network connections produces different impairments

Simulating spine loss by deleting network connections at random is an alternative to the weight decay approach used in this paper. However, the impairments produced in the agent by this approach have a different character to the impairments produced by weight decay, and are less depression-like. Specifically, this method of simulating spine loss does not produce the

anhedonia, increased discounting, or reversion to a simple survival strategy observed with weight decay. This is the case for 10%, 15%, 20%, and 30% connection loss. An example is shown below in figure S2.

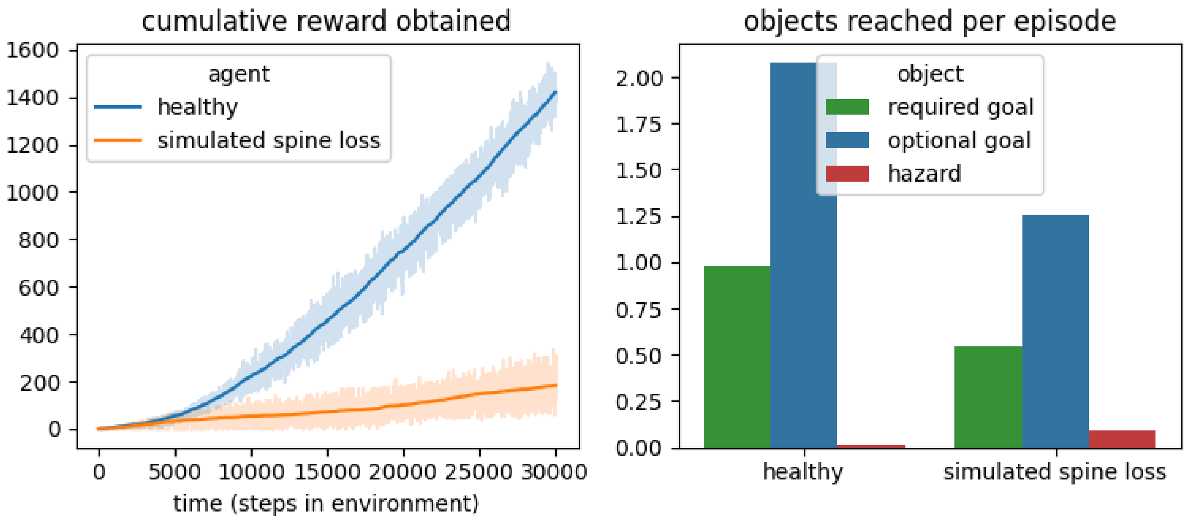

Figure S2, Agent performance results when simulating spine loss by deleting random connections. Compare paper figure 2: the agent with simulated spine loss is still impaired, but the impairment has a different character: the basic survival strategy of reaching the required goal is now also impaired.

Code

All code is available at: https://github.com/echalmers/blue_ai
